# THE ASSOCIATION OF PAIN LOCUS OF CONTROL WITH PAIN OUTCOMES AMONG OLDER ADULTS

**DOI:** 10.1093/geroni/igz038.979

**Published:** 2019-11-08

**Authors:** Shirley Musich, Shaohung Wang, Luke Slindee, Sandra Kraemer, Charlotte Yeh

**Affiliations:** 1 Optum, Ann Arbor, Michigan, United States; 2 Optum, Eden Prairie, Minnesota, United States; 3 UnitedHealthcare, Minneapolis, Minnesota, United States; 4 AARP Services, Inc., Washington, District of Columbia, United States

## Abstract

Pain locus of control (LOC) refers to perceived control over health and pain. LOC has ramifications on pain severity, disability and treatment. Our objective was to determine prevalence of 3 subscales of LOC among older adults with pain; and examine associations of scales on pain severity, opioids and physical function. The sample was identified from adults age≥65 with back pain, osteoarthritis or rheumatoid arthritis and minimum of 12-months medical and drug plan enrollment. Members were mailed a survey in May 2018 assessing positive resources, negative attributes and pain outcomes of pain. Opioid and other medications were determined from drug claims. The population was propensity weighted to adjust for survey non-response bias; weighted to generalize to those with diagnosed pain. Multivariate logistic regression was used to determine protective associations of positive resources with negative attributes on outcomes. Among respondents, 30% were internal LOC; 34% powerful other; 36% chance. In multivariate models, demographics, socioeconomics, health status, pain medications, resilience and social network, internal LOC was associated with lower pain severity, reduced opioid use and increased physical function. Magnitude of impact was comparable to resilience and diverse social networks. Powerful other LOC was partially protective with improved psychosocial attributes but poorer pain outcomes; chance LOC was associated with poor psychosocial/pain outcomes. Despite positive resources, depression and poor sleep maintained associations with pain outcomes. Internal LOC is a strong positive resource associated with improved pain. Multidimensional programs should include enhancing positive resources, including LOC, and focus on improving negative attributes to maximize pain management.

